# Regulation of Secondary Metabolism in the *Penicillium* Genus

**DOI:** 10.3390/ijms21249462

**Published:** 2020-12-12

**Authors:** Christelle El Hajj Assaf, Chrystian Zetina-Serrano, Nadia Tahtah, André El Khoury, Ali Atoui, Isabelle P. Oswald, Olivier Puel, Sophie Lorber

**Affiliations:** 1 Toxalim (Research Centre in Food Toxicology), Université de Toulouse, INRAE, ENVT, INP-Purpan, UPS, 31027 Toulouse, France; christel.hajjelassaf@hotmail.com (C.E.H.A.); Chrystian-Del-Carmen.Zetina-Serrano@inrae.fr (C.Z.-S.); nadia.tahtah@inrae.fr (N.T.); isabelle.oswald@inrae.fr (I.P.O.); sophie.lorber@inrae.fr (S.L.); 2 Institute for Agricultural and Fisheries Research (ILVO), member of Food2Know, Brusselsesteenweg 370, 9090 Melle, Belgium; 3 Centre D’analyse et de Recherche, Unité de Recherche Technologies et Valorisations Agro-Alimentaires, Faculté des Sciences, Université Saint-Joseph, P.O. Box 17-5208, Mar Mikhael, Beirut 1104, Lebanon; andre.khoury@usj.edu.lb; 4 Laboratory of Microbiology, Department of Life and Earth Sciences, Faculty of Sciences I, Lebanese University, Hadath Campus, P.O. Box 5, Beirut 1104, Lebanon; aatoui@ul.edu.lb

**Keywords:** *Penicillium*, secondary metabolism, regulation, virulence, control of gene expression, transcription factors

## Abstract

*Penicillium*, one of the most common fungi occurring in a diverse range of habitats, has a worldwide distribution and a large economic impact on human health. Hundreds of the species belonging to this genus cause disastrous decay in food crops and are able to produce a varied range of secondary metabolites, from which we can distinguish harmful mycotoxins. Some *Penicillium* species are considered to be important producers of patulin and ochratoxin A, two well-known mycotoxins. The production of these mycotoxins and other secondary metabolites is controlled and regulated by different mechanisms. The aim of this review is to highlight the different levels of regulation of secondary metabolites in the *Penicillium* genus.

## 1. Introduction

Studies have estimated the existence of at least 2.2–3.8 million fungal species on Earth, from which only around 10% have been isolated and described [1,2]. *Penicillium*, one of the most common fungi in a various range of habitats, has a worldwide distribution and a large economic impact on human life. This genus is of great importance in numerous and diverse fields, such as food spoilage, biotechnology, plant pathology, and medicine [3,4], and currently contains 483 accepted species [5]. Several of these species, classified as pre- and post-harvest pathogens, can lead to catastrophic decay in food crops, as described by Frisvad and Samson [6], Pitt and Hocking [7], and Samson et al. [8]. *Penicillium* can also produce a varied range of secondary metabolites, including several harmful mycotoxins [9], antibacterial [10,11,12,13,14] and antifungal compounds [15], immunosuppressants, and cholesterol-lowering agents [16,17,18,19]. The most iconic example of a drug of fungal origin is penicillin, the first antibiotic substance in history [20].

The biosynthesis of several secondary metabolites, such as mycotoxins, depends on several environmental cues including the substrate, pH, temperature, water activity, interrelationships with other microorganisms, and the interactions of these different factors in the natural environment [21,22,23].

**Figure 1:**
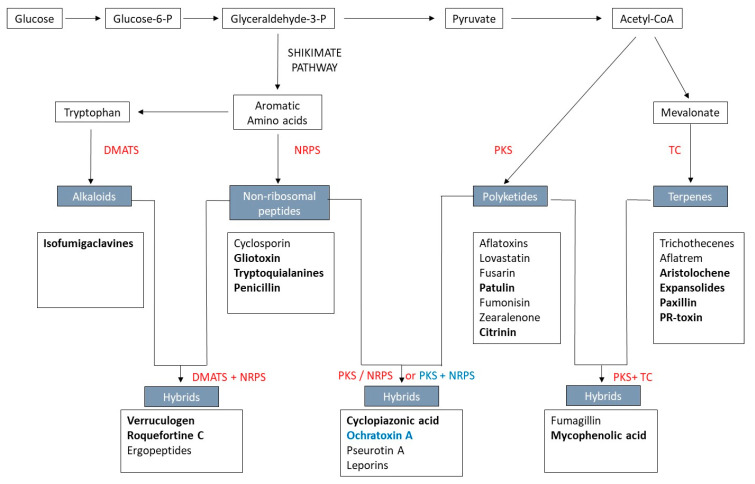
Biosynthetic pathways of secondary metabolites. In grey boxes, the typical backbone of secondary metabolites. In grey, the main mycotoxins produced by these pathways. In red, the enzymes associated with each pathway. In blue, separate PKS and NRPS are involved in ochratoxin A (OTA) biosynthesis; NRPS: non-ribosomal peptide synthetase, PKS: polyketide synthase, TC: terpene cyclase, DMATS: dimethylallyl tryptophan synthase. In bold, mycotoxins produced by *Penicillium* species.

Secondary metabolites are products of enzymatic cascades starting when backbone enzymes such as polyketide synthases (PKSs), non-ribosomal peptide synthetases (NRPSs), terpene cyclases (TCs), and dimethylallyl tryptophan synthases (DMATSs) catalyze, respectively, the rearrangement or condensation of simple primary metabolites, such as acetyl-CoA, amino acids, or isoprene units, resulting in more complex secondary metabolites [24]. Different metabolic pathways can lead to their formation (Figure 1). Fungal secondary metabolites are classified into five categories according to their structures and their precursors: polyketides, cyclic terpenes, non-ribosomal peptides, indole alkaloids, and hybrids (Figure 1) [25]. Other enzymes named tailoring enzymes are also needed and interfere in the catalysis of subsequent reactions in the biosynthetic pathways of mycotoxins. The structural diversity of mycotoxins results from the variety of chemical reactions (cyclization, aromatization, glycosylation, hydroxylation, methylation, acetylation, and epoxidation) involved in their biosynthesis [26] and leads to their broad spectrum of biological properties and functions. The combined involvement of backbone enzymes in the same biosynthesis pathway infinitely broadens this structural diversity of secondary metabolites. This diversity is also enriched by the infrequent existence of crosstalk between different biosynthetic pathways [27].

Enzymes are activated at the same time, and the newly synthesized intermediates are consecutively metabolized by the following enzymes. This phenomenon is possible due to the cluster organization of the genes encoding the enzymes involved in the biosynthesis of the metabolites in the same chromosomal region. These genes are often co-activated by a specific transcription factor (TF) located within the clusters [28]. Based on bioinformatics analysis and other studies, it was proven that fungal genomes exhibit different and numerous predicted secondary metabolite clusters. A recent review estimated the number of fungal biosynthetic gene clusters (BGCs) at several million [29]. For the two well-known genera of *Aspergillus* and *Penicillium* alone, which contain 446 and 483 species, respectively [5], the number of non-redundant clusters is approximately 25,000. In filamentous fungi, the activation of specific TFs and the resulting production of fungal secondary metabolites is controlled at a higher hierarchical level by global TFs. Understanding the mechanisms underlying mycotoxin biosynthesis contributes to defining/identifying strategies or mechanisms to regulate them and reduce their production [30].

Numerous studies have focused on regulators impacting the formation of secondary metabolites in *Aspergillus*, *Penicillium*, and *Fusarium*, but few reviews have explored the complex and multi-layered regulation of fungal secondary metabolism [31,32,33,34]. While several excellent articles reviewed the different regulatory mechanisms known for *Aspergillus* [29,35,36,37], this review aims to deepen the understanding of the regulation of secondary metabolism in *Penicillium* and highlight all the regulatory mechanisms that can occur.

## 2. Regulation of Secondary Metabolism

For the synthesis of any secondary metabolite, the regulation of its cluster involves a number of factors for activation or repression. This regulation occurs at different levels. Most secondary metabolite clusters have genes encoding TFs that act directly on all other genes located within the cluster. Expression of these internal regulators also depends on other, more global TFs encoded by genes unrelated to the BGCs, which are themselves under the control of different physiological and/or environmental stimuli. An adaptation to a specific environment may also result in the biosynthesis of a certain secondary metabolite. This biosynthesis is connected and regulated by different signaling transduction pathways. Finally, epigenetic regulation, including modification of the chromatin and nucleosome structure, can yield transcriptional control and impact secondary metabolite synthesis extensively [38]. In the following section, the different regulatory systems studied in *Penicillium* will be discussed.

### 2.1. Specific Transcription Factors/Cluster-Specific Regulators

**Figure 2:**
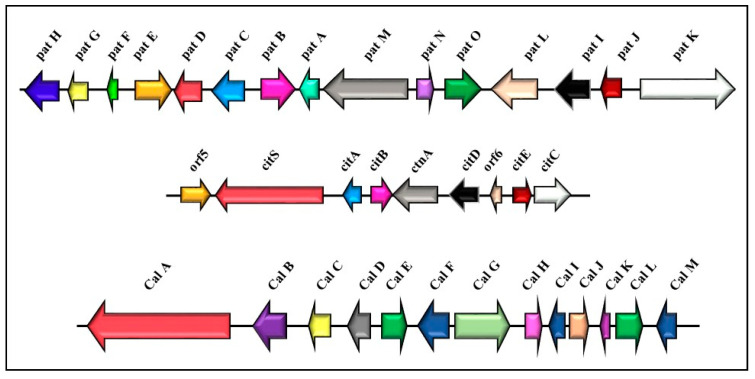
Gene clusters of the patulin biosynthesis pathway (the first one at the top) (15 genes, 40 kb) [39] and the citrinin biosynthesis pathway (the middle group) (nine genes, 22 kb) [40,41] in *Penicillium expansum*; cluster of the calbistrin biosynthesis pathway (the third one at the bottom) (13 genes, 35kb) in *Penicillium decumbens* [42].

Gene clusters involved in secondary metabolite biosynthesis often include a gene encoding a TF that specifically acts and modulates the expression of the other genes in that cluster (e.g., *patL*, *calC*, and *ctnA* in patulin, calbistrin, and citrinin biosynthetic pathways, respectively). This gene has a switching role within the cluster (Figure 2). The TFs regulate gene expression by binding specifically to the promoters of the genes involved.

Several studies comparing TF sequences have shown that these TFs can be classified into different families based on the similarities in their protein sequences. We can distinguish between zinc finger proteins, proteins called helix-turn-helix, and leucine zippers [43]. Nevertheless, almost 90% of the potential gene clusters involved in the synthesis of fungal polyketides belong to the family of zinc finger TFs (Cys_2_His_2_, Cys_4_, or Zn(II)_2_Cys_6_) [43,44,45]. Proteins of the Zn(II)_2_Cys_6_ family are found exclusively in fungi and yeasts [46], and the C_6_ type zinc finger DNA binding protein motif (Cys_6_) is frequently encountered in TFs. Cys_6_ has been identified on more than 80 proteins found mainly in fungi [43] and is generally considered a transcriptional activator (Table 1). Only in *Saccharomyces cerevisiae* are the zinc finger proteins (ARGR2, LEU3, and UME6) activators and repressors [47,48,49,50]. Subsequently, the number of proteins belonging to the Zn(II)_2_Cys_6_ family has increased significantly due to the number of fungal genomes that have since been sequenced. Numerous examples of Zn(II)_2_Cys_6_ TFs identified as being involved in the secondary metabolism of fungi genera other than *Penicillium* have been largely described in the literature. As examples, we can quote AflR (aflatoxins), Bik5 (bikaverin), and CtnA (citrinin) for *Aspergillus*, *Fusarium*, and *Monascus*, respectively (Table 1). 

Gliotoxin, a secondary fungal metabolite belonging to the class of epipolythiodioxopiperazines (ETPs) and characterized by the presence of a sulfur-bridged dioxopiperazine ring [91], is produced by some *Aspergillus* and *Penicillium* species, such as *Penicillium lilacinoechinulatum* [92], a strain of this species was misidentified as *Penicillium terlikowskii* in a study by Waring et al. [92,93]. Within its cluster, a Zn(II)_2_Cys_6_ finger transcription regulator, GliZ, was identified to be responsible for gliotoxin induction and regulation. A mutation of the *gliZ* (∆*gliZ*) gene in *Aspergillus fumigatus* resulted in the loss of gliotoxin production, while overexpression of *gliZ* increased the production of gliotoxin [94,95]. In *P. lilacinoechinulatum*, a homologous gene is present in the genome, but the heterologous complementation of the *A. fumigatus* ∆*gliZ* mutant with Pl*gliZ* failed to restore gliotoxin production [58]. The *mlcR* gene encoding a putative 50.2-kDa protein characterized by a Zn(II)_2_Cys_6_ DNA-binding domain was shown to be involved in the regulation and biosynthesis of ML-236B (compactin) in *Penicillium citrinum* [96].

Another gene encoding PatL, a specific TF in *Penicillium expansum*, was shown to affect patulin production [85]. The protein encoded by this gene has two conserved domains, one of which encodes a Cys_6_ DNA binding site and the other of which was found in the TFs of the superfamily of zinc finger TFs. Orthologous genes of *patL* involved in the patulin metabolic pathway were found in other filamentous fungi genomes, such as *Penicillium griseofulvum*, *Penicillium paneum*, *Penicillium vulpinum*, *Penicillium carneum*, *Penicillium antarcticum* [97], and *Aspergillus clavatus* [98]. Sometimes, BGCs such as the sorbicillin gene cluster can contain two genes encoding TFs. In this example, SorR1, a Zn(II)_2_Cys_6_ factor, acts as an activator for the expression of all genes located within the cluster. The second zinc finger TF (SorR2) controls the expression of the *sorR1* gene [86]. Few cases of TFs belonging to the basic leucine zipper (bZIP) family have been reported to act as specific TFs of secondary metabolite pathways. These TFs include ZEB2, SimL, and OtaR1. The latter TF is present in the OTA cluster in *Aspergillus ochraceus*, *Aspergillus westerdijkiae*, *Aspergillus carbonarius*, and *Penicillium nordicum*. Its inactivation in *A. ochraceus* leads to the complete inhibition of OTA production [63]. SimL regulates the production of the well-known immunosuppressant drug cyclosporine [62]. The transcripts of genes located within the zearalenone gene cluster were not detected when the *zeb2* gene encoding bZIP was deleted [61,99]. *TqaK*, another gene encoding a bZIP protein, was reported to be located inside the tryptoquialanine gene cluster in *Penicillium aethiopicum*. The deletion of *tqaK* led to tryptoquialanine production equal to only one-twentieth that of the parental strain [87]. An orthologous gene is also present in the genome of *Penicillium digitatum,* another tryptoquialanine-producing species. Thus far, OtaR1 and TqaK are the only bZIP proteins identified to be directly involved in secondary metabolite biosynthesis in *Penicillium*.

### 2.2. Environmental Signals and Associated Regulators

**Figure 3:**
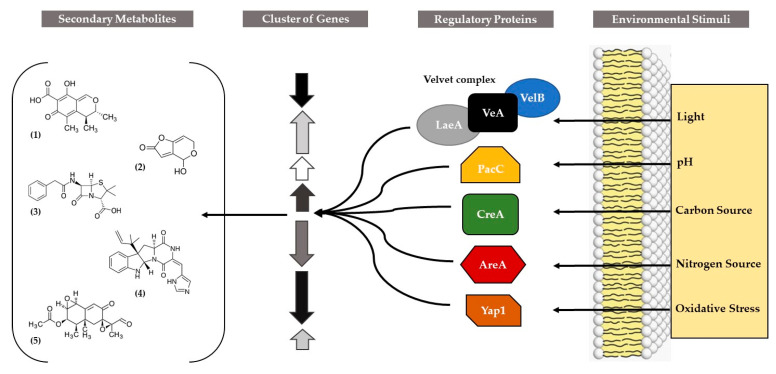
Global regulatory proteins involved in the regulation of gene clusters involved in the production of various secondary metabolites in *Penicillium* (1) citrinin, (2) patulin, (3) penicillin G, (4) roquefortine C, and (5) PR-toxin, adapted from Brakhage [45].

In the previous section, we reviewed specific TFs described in *Penicillium* that are cluster-specific. However, numerous regulatory elements affected by environmental cues modulate the expression of fungal secondary metabolite clusters and do not reside within the cluster itself. They are considered to be global regulators (Figure 3). Among them, CreA, AreA, Nmc, PacC, Skn7, Yap1, VeA, LaeA, BrlA, PcRFX1, PcFKH1, Pcz1, and NsdD have been mentioned and are discussed in the following.

In the fungal kingdom, the synthesis of secondary metabolites is often a response to environmental or ecological changes and is dependent on the developmental stage of the producing species. The activation of a biosynthetic pathway is influenced by the composition of the substrate on which the fungus grows—in particular, the carbon source and the nitrogen source. Glucose and other assimilable sugars can suppress secondary metabolite pathways mediated by **CreA**, a protein displaying two Cys_2_His_2_ zinc finger domains. For example, the biosynthesis of penicillin in *Penicillium chrysogenum* was shown to be largely regulated by glucose, sucrose and, to a lesser extent, by other sugars (maltose, fructose, and galactose). Cepeda-García et al. [100] showed clear evidence of the involvement of the CreA factor in the catabolic repression of penicillin biosynthesis and the expression of the *pcbAB* gene, encoding the first enzyme of the penicillin pathway in *P. chrysogenum*. The authors applied an RNAi strategy attenuating *creA* gene expression. Transformants expressing small interfering RNAs for *creA* showed greater production of penicillin. By contrast, a recent study showed that the deletion of *creA* in *P. expansum* strains leads to the absence of patulin production in apples [101], although expression of the *pat* genes is increased. Regarding the nitrogen source, a similar regulatory mechanism called nitrogen metabolite repression exists in Ascomycetes. For instance, a concentration of ammonium above 40 mM caused a repression in the expression of *uidA*, a promoterless gene for β-glucuronidase in *Escherichia coli*, when fused to the promoters of *pcbAB* (*acvA*) and *pcbC*, two genes encoding the two first enzymes of the penicillin pathway in *P. chrysogenum* [102]. In *P. griseofulvum* (formerly *P. urticae*)*,* the production of patulin was also affected when ammonium ions were added to the culture medium [103]. On the other hand, the presence of 30 mM ammonium chloride results in a significant decrease in isoepoxydon dehydrogenase (*idh*) and 6-methylsalicylic synthase (*6-msas*) transcripts, key genes in the pathways of patulin biosynthesis [104,105]. This nitrogen metabolite repression is mediated by **AreA**, a Cys_2_Cys_2_-type zinc finger TF [31]. This regulatory factor binds to the intergenic region of *acvA*-*pcbC* [106] in response to nitrogen and mediates the regulation of penicillin biosynthesis in *P. chrysogenum* [107]. The *idh* (*patN*) and *6-msas* (*patK*) genes interact with the NrfA protein, an orthologue of the AreA protein in *P. griseofulvum*, through several putative GATA sites located on their promoter. The *nmc* gene, encoding the AreA orthologous factor, has been characterized in *Penicillium roqueforti*. This protein, which displays at least 94% identity with that of homologous fungal proteins (AreA in *Aspergillus*) [108], is induced and upregulated by nitrogen starvation, but no data regarding its impact on *P. roqueforti* secondary metabolites have already been published. 

Another well-known environmental stimulus that induces or represses the secondary metabolism in filamentous fungi is the pH of the substrate. This regulation is mediated by **PacC**, the key factor of pH fungal regulation [109]. This TF displays three putative Cys_2_His_2_ zinc fingers [110]. In the genus *Aspergillus*, a neutral to alkaline extra-cellular pH is required for the activation of PacC via two proteolytic steps [111]. These steps are mediated by the *pal* (*palA*, *palB*, *palC*, *palF*, and *palI*) pathway [109]. The final mature form of this protein activates the expression of genes expressed under alkaline conditions and, by contrast, represses the transcription of genes expressed under acidic conditions. Many examples of PacC’s involvement in the regulation of biosynthetic pathways in *Aspergillus* or *Fusarium* species have been reported in the literature [65,112,113]. Suárez and Peñalva [114] showed that *Penicillium pacC* transcript levels were higher under alkaline than acidic growth conditions and elevated in later stages of growth. The level of the *pcb* transcripts followed the same trend, leading to increased production of penicillin under alkaline pH. Barad et al. [115] also studied the link between ammonia accumulation, the activation of *pacC*, and the synthesis of patulin in *P. expansum*. The authors concluded that an accumulation of ammonia during nutritional limitation in *P. expansum* could lead to a modification of the ambient environmental pH, a signal for the activation of *pacC*, as well as other alkaline induced genes leading to an accumulation of secondary metabolites, such as patulin.

Barad et al. [116] analyzed the role of PacC in the regulation of D-gluconic acid (GLA) production and patulin accumulation in *P. expansum*. On the one hand, their results showed that GLA production plays a role in the activation of patulin production. On the other hand, this study, based on the characterization of *pacC*-RNAi mutants of *P. expansum*, concluded that PacC plays a key role in the regulation of GLA accumulation via the transcriptional regulation of *gox2*, the most important gene involved in GLA production in *P. expansum*. This regulation of GLA production through PacC largely affects patulin accumulation in the mutants. A recent publication reported that the production of patulin is completely inhibited in the null mutant Pe∆*pacC* strain when grown at pH > 6.0 [117]. In *P. digitatum*, PacC was reported to regulate the expression of genes encoding polygalacturonase PG2 and pectin lyase PNL1, enzymes both involved in the degradation of the citrus cell wall [118]. 

Osmotic and oxidative stress are considered to be other environmental cues to which filamentous fungi should respond in order to survive. Most of the relevant knowledge comes from the yeast *S. cerevisiae* and the fungal genus *Aspergillus*. **Skn7**, a TF involved in the osmotic and oxidative stress responses in *S. cerevisiae* [119], has also been identified in *Talaromyces* (formerly *Penicillium*) *marneffei*. The gene *skn7* from the latter was used to complement a *skn7*-disrupted strain of *S. cerevisiae* and seemed to be involved in the oxidative stress response in the yeast [120]. This result indicates the highly conserved nature of *skn7* between the two organisms. Montibus et al. [121] suggested that *skn7* could be involved in the regulation of fungal secondary metabolism. A recent study seemed to confirm this hypothesis since the deletion of Af*skn7* resulted in a drastic decrease in aflatoxin B1 production in *Aspergillus flavus* [122]. **Yap1**, another TF, coordinates the interplay between oxidative stress and secondary metabolism. In *Aspergillus parasiticus*, the deletion mutant ∆*yap1* exhibited an increase in aflatoxin production [123]. The same team later reported that the suppression of the *yap1* orthologous gene led to increased OTA accumulation in *Aspergillus ochraceus* [124]. In *T. marneffei*, the mutant ∆*yapA*, a *yap1* orthologous gene, was found to be sensitive to oxidative chemicals such as H_2_O_2_ or menadione and featured growth, germination, and conidiation delays [125]. For the genus *Penicillium*, the only works on orthologues Skn7 and Yap1 are mentioned above; their roles in the secondary metabolism of *T. marneffei* have not yet been investigated. 

The development of filamentous fungi and their ability to produce secondary metabolites is largely influenced by light, as well. A velvet complex has been described in *Aspergillus nidulans*, and the **VeA** (velvet A) factor has been widely studied, as well as many proteins that seem to interact with it, such as VelB (velvet-like B), VosA (viability of spores A), VelC (velvet-like C), and the non-velvet protein **LaeA** (loss of *aflR* expression A), a methyltransferase involved in chromatin remodeling [126]. Depending on the fungal species, VeA is involved in different physiological processes, such as development, asexual and sexual reproduction, secondary metabolism, and virulence. The regulation mediated by this factor depends particularly on light. VeA was first characterized in *A. nidulans*, whose gene encodes a protein of 573 amino acids with a conserved domain at the N-terminus [127] and a nuclear localization sequence (NLS) [128]. At its C-terminus, a PEST domain (rich in proline (P), glutamic acid (E), serine (S), and threonine (T)) is present [129]. This PEST domain is also found in VeA orthologous proteins in *A. parasiticus*, *A. fumigatus*, and *Neurospora crassa* [130]. 

Stinnett et al. [128] studied the intracellular localization of VeA. This study demonstrated that this localization is dependent on light. In the dark, VeA is mainly located in the nucleus, whereas in the presence of light, VeA is mainly found in the cytoplasm. In the *veA1* mutant [131], VeA is mostly found in the cytoplasm independently of light. In this mutant, the presence of a mutation on the transcription initiation codon led to a truncated protein where the first 36 amino acids were missing and, therefore, did not have a functional NLS, thus explaining the cytoplasmic localization of VeA. In the same study, it was demonstrated that the transfer of VeA into the nucleus depends on the importin α KapA and that a functional NLS is essential to allow the interaction of these two proteins.

To identify the proteins interacting with VeA, Bayram et al. [127] used the Tandem Affinity Purification (TAP) technique from a strain of *A. nidulans* expressing a VeA protein coupled to a TAP-tag at the C-terminus. In the dark, the proteins VelB, LaeA, and importin α KapA interact with VeA. Conversely, only VelB interacts with VeA in the presence of light. Using the yeast two-hybrid technique, these analyses confirmed the interactions of VeA–VelB and VeA–LaeA; however, no interaction was demonstrated between LaeA and VelB, suggesting that VeA acts as a bridge between these two proteins. In addition, fluorescence assays showed that the VeA–LaeA interaction occurs in the nucleus, while VeA and VelB interact in the nucleus and the cytoplasm. LaeA is located in the nucleus, and its interaction with VeA is nuclear. VelB must, therefore, be able to enter the nucleus despite the absence of NLS in its sequence. Bayram et al. [127] demonstrated that VeA helps VelB to enter the nucleus to form the velvet complex.

**Figure 4:**
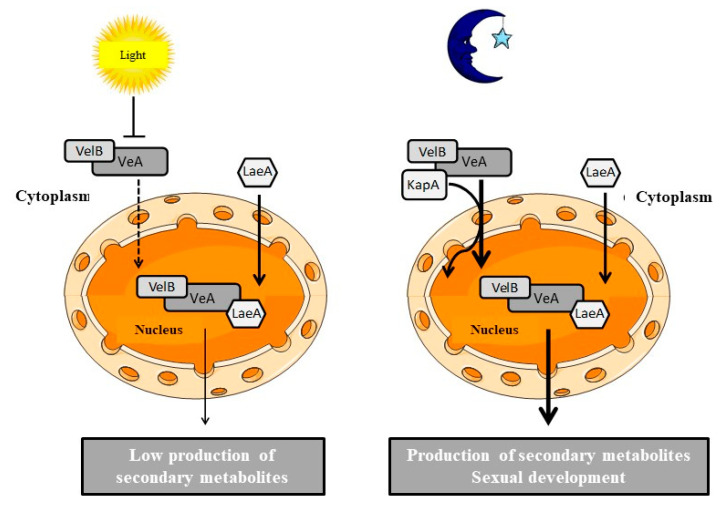
Operating model of the velvet complex in *Aspergillus nidulans* adapted from Bayram et al. [127]. In the presence of light, VeA is retained in the cytoplasm (-----), and LaeA has low activity. In the dark, VeA coupled to VelB is transported in the nucleus by the importin α KapA (——), and the velvet complex is formed with LaeA to activate the production of secondary metabolites and sexual development.

The results obtained in the various studies allowed Bayram et al. [127] to propose a mechanism (Figure 4) that coordinates the regulation of sexual development and the production of secondary metabolites in *A. nidulans*. In the dark, the VelB/VeA/LaeA complex controls and induces the epigenetic activity of LaeA, which consequently controls the expression of the genes of the clusters responsible for the synthesis of the secondary metabolites. In the presence of light, this interaction decreases because VeA is retained in the cytoplasm, and LaeA has low activity.

Despite its strong conservation among different fungal species, VeA has different roles, reflecting the diversity of fungal development patterns. Therefore, *veA* has a role in the regulation of secondary metabolism. The expression of genes involved in the synthesis of secondary metabolites is affected by VeA [132,133,134,135]. Kato et al. [132] demonstrated that in *A. nidulans*, VeA regulates the expression of genes involved in sterigmatocystin synthesis. Indeed, VeA is necessary for the expression of *aflR*, which encodes the TF specific to the biosynthetic pathway of this mycotoxin [136]. Similarly, the *veA* gene is required for the transcription of *aflR* and *aflJ*, another gene coding for a TF, also located within the aflatoxin/sterigmatocystin cluster in *A. flavus* [137,138]. Other studies revealed that VeA is needed for the synthesis of other secondary metabolites, such as cyclopiazonic acid and aflatrem in *A. flavus* [133], penicillin in *A. nidulans* [132], or trichothecenes in *Fusarium graminearum* [139]. In this last study, FgVe1 was shown to be a positive regulator of the virulence of *F. graminearum*. In *Fusarium verticillioides*, FvVe1 is necessary not only for the production of fumonisins but also for the infection of corn plants by the fungus [140]. In *P. chrysogenum*, *veA* controls penicillin biosynthesis [141]. Recently, it was shown that the disruption of *veA* in *P. expansum* quasi-totally altered patulin and citrinin production when the fungus was grown on the usual mycological media (Malt Extract Agar and Potato Dextrose Agar). This decrease in production is explained by a drastic decrease in the expression of patulin and citrinin genes [142]. This finding was confirmed in vivo, as no patulin was detected when the null mutant was developed in apples. In the same study, an analysis of the impact of VeA on the expression of all secondary metabolism backbone genes in *P. expansum* was performed from the genome of the d1 strain, including PKS, NRPS, terpene synthase, and DMATS genes. The expression analysis showed a positive or negative regulation of 15/35 backbone genes and supports the hypothesis that *P. expansum*’s secondary metabolism is modulated by the transcriptional regulator factor VeA. In a recent study, Li et al. [143] assessed the involvement of the proteins VeA, VelB, VelC, and VosA, belonging to the velvet family, in the regulation of patulin biosynthesis in *P. expansum*. The absence of VeA and VelB blocked the production of the mycotoxin, whereas the absence of VelC caused a drastic decrease in patulin production. In contrast, deletion of the *vosA* gene had no effect on the capacity of the fungus to synthesize patulin. These findings suggest the lack of involvement of VosA in the biosynthesis of patulin in *P. expansum* in contrast to the other three proteins (VeA, VelB, and VelC) of the velvet complex.

Baba et al. [144] also showed through gene deletion that *veA* plays critical roles in the production of the hypocholesterolemic lovastatin analogue compactin (ML-236B) in *P. citrinum* by controlling the expression of *mlcR*, the pathway-specific activator gene for compactin biosynthesis.

It was also shown that different components of the velvet complex may play opposite roles in the regulation of secondary metabolism. In *P. chrysogenum*, PcVelC, together with the velvet PcVeA (orthologue of VeA in *P. chrysogenum*) and the methyltransferase PcLaeA, induced penicillin production, and, in contrast, PcVelB acted as a repressor [141,145].

Under the conditions tested by Kosalkova et al. [146], LaeA controlled some secondary metabolism gene clusters in *P. chrysogenum*. Its overexpression resulted in a four-fold increase in *pcbC* and *penDE* expression, leading to a 25% increase in gene expression in penicillin biosynthesis, while its suppression significantly reduced the expression of these genes. In contrast, the absence of an expression level difference (∆*laeA* vs. wild type (WT)) for the *rpt* gene involved in the second step of the roquefortine biosynthetic pathway suggests that Pc*laeA* does not regulate the biosynthesis of roquefortine C. The regulation of the secondary metabolism of *P. expansum* by *laeA* was investigated from two cultures on different media [147]. Of the 54 backbone genes examined, many appeared to be positively regulated by *laeA*, such as those involved in the biosynthesis of roquefortine C, an unknown ETP-like metabolite, and patulin. In *Penicillium oxalicum*, it has been shown that the putative methyltransferase LaeA controls, among other things, the expression of some secondary metabolic gene clusters [148]. However, the cluster predicted to be involved in roquefortine C/ meleagrin/oxaline biosynthesis was not affected by the suppression of the *laeA* gene in *P. oxalicum*. The difference observed between these studies regarding *laeA* regulation of the genes involved in the biosynthesis of roquefortine C could be due to the species used and the medium tested.

Zhu et al. [149] demonstrated the role of *laeA* in secondary metabolism regulation, conidial production, and stress responses in *P. digitatum*. The deletion of Pd*laeA* resulted in decreased expression of various secondary metabolite gene clusters, including the *Tq* cluster involved in tryptoquialanine biosynthesis. Deletion of this gene also affected the expression of several regulators of conidiation, including BrlA. A comparison between the WT and the null mutant Pd∆*laeA* strains revealed increased sensitivity of the null mutant strain under alkaline conditions. The loss of Pd*laeA* had no significant effect on the virulence of the null mutant strain. This work showed the involvement of *laeA* in the biosynthesis of several secondary metabolites, as well as the development and the adaptation of *P. digitatum* to its environment.

Yu et al. [150] showed that the overexpression of LaeA in the *Penicillium dipodomyis* marine-derived strain YJ-11 leads not only to morphological but also metabolic changes. Overexpression mutants displayed the ability to produce several sorbicillinoids, two of which are new compounds, as well as four known sorbicillin analogues. These results indicate that LaeA plays a key role in the activation of cryptic genes that are silent under normal *laeA* expression.

Kumar et al. [151] showed the effects of the intrinsic factors of apples in modulating patulin accumulation and on *laeA* and *pat* gene expression in apples colonized by *P. expansum*. The authors used two apple varieties, Golden Delicious and Granny Smith, which have similar total soluble solid value profiles at the time of ripening but different pH values and malic acid concentrations. These factors differentially affected the expression of LaeA along with the expression of the patulin cluster genes and, therefore, patulin accumulation. To understand the complexity of these interactions, in vitro studies were performed. These studies proved that sucrose and malic acid concentrations and pH are all involved, in association with chlorogenic acid and epicatechin, in a complex interaction system that modulates the regulation and production of patulin.

*Penicillium brocae* HDN-12-143 is a fungus isolated from marine sediments that has strong potential for the biosynthesis of secondary metabolites. Wang et al. [152] studied the effect of overexpression of the *laeA* gene on the secondary metabolism of *P. brocae*. This overexpression revealed that four compounds could be isolated, including fumigatin chlorohydrin and a new polyketide compound, iso-fumigatin chlorohydrin. In summary, the results indicate that LaeA can suppress or activate the expression of gene clusters and that its overexpression can induce the production of new secondary metabolites.

In *Aspergillus*, VeA is responsible for the activation or repression of general genes such as *brlA* [134,153]. **BrlA** is a C_2_H_2_-type zinc finger TF which is part of the central regulatory pathway (CRP) controlling the expression of genes specific to asexual reproduction. The conformation of *brlA* is complicated and comprises two overlapping transcription units, *brlAα* and *brlAβ* [154]. Expression of the *brlA* gene was studied in *P. oxalicum* strains, initially identified as *Penicillium decumbens,* by Qin et al. [155], and the expression levels of 7/28 gene clusters of secondary metabolism were regulated in a ∆*brlA* deletion strain. The cluster involved in the roquefortine C/meleagrin/oxaline biosynthetic pathway was downregulated. In a *P. chrysogenum brlA*-deficient mutant, the production of penicillin V was not affected, whereas a reduction of almost 99% was determined via HPLC analysis accompanied by a drastic downregulation of the expression of penicillin biosynthetic genes in a *stuA*-deficient strain [156]. Moreover, the deletion of *laeA* reduced the conidiation in *P. oxalicum*, and the expression of *brlA* was downregulated [148]. A recent study in *P. expansum* showed that the *brlA* gene not only affects the stage of conidiation of the fungus but also affects the biosynthesis of secondary metabolites. Zetina-Serrano et al. [157] showed that the suppression of *brlA* results in a strain devoid of conidia and that the production of communesins and derivatives was drastically decreased, whereas the production of chaetoglobosins and derivatives increased. Neither patulin nor citrinin production was affected by the suppression of *brlA*.

**PcRFX1** is a TF that was characterized in *P. chrysogenum* by Domínguez-Santos et al. [158]. PcRFX1 is the orthologue of the regulatory proteins CPCR1 and RFxA in *Acremonium chrysogenum* and *T. marneffei*, respectively. Knockdown and overexpression techniques of the Pc*rfx1* gene have proven that PcRFX1 regulates *pcbAB*, *pcbC,* and *penDE* transcription and thereby controls penicillin biosynthesis. PcRFX1 was also suggested to be involved in the control of the pathways of primary metabolism.

**PcFKH1**, another TF of the winged-helix family, also positively regulates penicillin biosynthesis in *P. chrysogenum* by binding to the *pcbC* promoter, interacting with the promoter region of the *penDE* gene and controlling other genes such as *phlA* and *ppt* encoding phenylacetyl CoA ligase and phosphopantetheinyl transferase [159].

The ***pcz1*** gene (*Penicillium* C6 zinc domain protein 1), encoding a Zn(II)2Cys6 protein and controlling the growth and development processes of the fungus, has also been described in *P. roqueforti*. It was suggested to participate in the physiological processes in this fungus and plays a key role in regulating its secondary metabolism [160,161]. The silencing of *pcz1* in *P. roqueforti* resulted in the downregulation of the *brlA*, *abaA,* and *wetA* genes of the CRP [160]. In *pcz1* downregulated strains, the production of the metabolites roquefortine C, andrastin A, and mycophenolic acid was severely reduced; however, when *pcz1* was overexpressed, only mycophenolic acid was overproduced, and levels of roquefortine C and andrastin A were decreased [161]. 

Finally, the Pox*nsdD* gene of *P. oxalicum* was characterized by He et al. [162]. This gene is an orthologue of the ***nsdD*** gene (initially isolated in *A. nidulans*) encoding a GATA-type zinc finger TF that was proven to be involved in the production of secondary metabolites. In the PoxΔ*nsdD* strain, the 230 differentially expressed genes identified covered 69 putative BGCs. Among them, 11 were predicted to produce aspyridone, emericellin, citrinin, leucinostatins, roquefortine C/meleagrin, beauvericin, cytochalasin, malbrancheamide, and viridicatumtoxin.

### 2.3. Signal Transduction Pathways

In general, fungi present a very dynamic and structured cell wall. During the cell cycle, organisms need to adapt quickly to changes under environmental conditions and imposed stresses and thus regulate the composition and structural organization of their cell wall [163,164,165]. All these factors influence the biosynthesis of secondary metabolites in the fungus. Numerous signaling pathways activate and regulate the growth and differentiation of filamentous fungi and initiate secondary metabolite biosynthesis under specific conditions. These signaling pathways sense and transduce signals external to TFs that, in turn, activate the expression of genes that could be involved in the biosynthesis of certain secondary metabolites. The cyclic adenosine monophosphate (cAMP)/protein kinase A (PKA), calcineurin/calmodulin, TOR, and mitogen-activated protein kinase are the most studied pathways [34]. The production of many secondary metabolites has been associated with one of these transduction signals and specific active molecules. Among the different signaling pathways listed, we focus on those that affect only the secondary metabolism of *Penicillium*, starting with the cAMP pathway.

#### 2.3.1. cAMP Pathways

Heterotrimeric G proteins are considered to be important components of these signal transduction pathways. They can integrate a variety of signals and then transduce them to downstream signaling cascades. Most filamentous fungi have three Gα proteins belonging to classes I, II, or III [166]. Gα subunits belonging to class I are involved in many aspects related not only to the development of the fungus or its pathogenicity but also its secondary metabolism, which is not the case for Gα subunits of classes II and III. The deletion of genes encoding class II Gα proteins showed negligible effects on fungal metabolism [167,168], while those of class III are involved in fungal development and pathogenicity [169,170,171]. Alterations have been observed in the secondary metabolism of different fungi, including *P. chrysogenum* [172] and *T. marneffei* [173]. The *pga1* gene, encoding subunits of subgroup I Gα protein in *P. chrysogenum*, has been shown to affect the production of three secondary metabolites: penicillin, chrysogenin, and roquefortine C. The deletion of *pga1* induces a decrease in the production of roquefortine C and penicillin by regulating the expression of *pcbAB*, *pcbC*, and *penDE*, the three structural biosynthetic genes of the penicillin cluster. Chrysogenin biosynthesis is enhanced, and roquefortine and penicillin biosynthesis is upregulated by the presence of a dominant activating *pga1* (G42R) allele or a constitutively active Pga1 [172]. Based on a proteomic analysis, Carrasco-Navarro et al. [174] suggested that Pga1 signaling affects penicillin biosynthesis by acting on the primary metabolism pathways that are also involved in cysteine, ATP, and NADPH biosynthesis. They also propose a model for the Pga1-mediated signal transduction pathway.

#### 2.3.2. The Osmostress Response Pathway

Usually, inhibition of the HOG (high osmolarity glycerol) signaling pathway negatively affects the production of metabolites; in other words, challenging osmotic conditions activate the cascade of the HOG MAP kinase signal, thereby activating several osmo-regulated genes or downstream TFs by phosphorylation. In their study, Stoll et al. [175] showed that NaCl induced production of OTA in correlation with the phosphorylation status of the HOG MAP kinase in *P. nordicum* and *Penicillium verrucosum*. The activation of HOG phosphorylation and the concomitant OTA biosynthesis suggest a link between the two processes and that this regulation may be mediated by the HOG MAP kinase signal transduction pathway. This was confirmed by inactivating the *hog* gene in *P. verrucosum*, making the fungus unable to produce OTA under high NaCl conditions. The biosynthesis of citrinin, another *P. verrucosum* toxin, was not affected. This could be explained by the subsequent work of Schmidt-Heydt et al. [176], which showed the impact of high oxidative stress conditions on citrinin biosynthesis. Indeed, by increasing Cu^2+^ concentrations in a growth medium, *P. verrucosum* shifts the biosynthesis of its secondary metabolism from OTA to citrinin. Increasing amounts of external cAMP reduce citrinin biosynthesis depending on the concentration chosen and suggest that citrinin biosynthesis is regulated by a cAMP/PKA signaling pathway.

### 2.4. Epigenetic Regulation

As previously discussed, *Penicillium* species, including other fungi, produce a set of bioactive secondary metabolites that are not essential to their survival. Genes for biosynthesis and the regulation of secondary metabolites in fungi are not evenly distributed over the genomes and tend to be sub-telomerically located [177]. The manipulation of global epigenetic regulators has contributed to the study of many unknown secondary metabolites, and many histone modifications have been associated with the regulation of secondary metabolism gene clusters [178,179]. The epigenetic phenomena that can occur are reversible, and many changes in the gene expression levels of the fungus do not alter the DNA sequence and can occur throughout the fungus life cycle. Fungal epigenetic regulation involves mainly histone modifications, such as methylation, acetylation, and sumoylation. Histone proteins are the primary protein components of chromatin and, through their modifications, regulation can be limited to a specific region of the chromosome and, therefore, affect some genes. This supports the advantage of grouping secondary metabolism genes into clusters. The first involvement of the epigenetic regulation of secondary metabolites described in the literature was that of the *A. nidulans* histone deacetylase coded by *hdaA,* an orthologue of the histone deacetylase *hdaA1* gene of *S. cerevisiae*. Deletion of this gene caused the activation of two secondary metabolite gene clusters. In the same paper, treatment of the *P. expansum* culture with trichostatin A, a histone deacetylase (HDAC) inhibitor, resulted in the overproduction of several non-determined metabolites [179].

In *P. chrysogenum*, *hdaA* appears to be a key regulator of the secondary metabolism of the fungus. Deletion of *hdaA* induced a significant effect on the expression of numerous PKS and NRPS-encoding genes. A downregulation of the NRPS encoded gene associated with the BGC of chrysogine was also observed. This observation was confirmed by Ding et al. [180]. In parallel, transcriptional activation of the BGC of sorbicillinoids occurs, which is associated with the detection of a new compound produced only under these conditions. These results obtained by Guzman-Chavez et al. [181] suggest the existence of crosstalk between BGCs. In a recent study, the disruption of *hdaA* led to an upregulation of the meleagrin/roquefortine C biosynthesis gene cluster, accompanied by higher meleagrin production [180].

Akiyama et al. [182] investigated the involvement of *clr3* in *Penicillium brasilianum* physiology. **Clr3** is a homologue of the class 2 histone deacetylase *hda1* in *S. cerevisiae*. On the one hand, the deletion of *clr3* resulted in decreased fungal growth under oxidative stress conditions. In addition, various secondary metabolites, such as austin-related meroterpenoids, brasiliamides, cyclodepsipeptides, and mycotoxins, including verruculogen and penicillic acid, were downregulated in the null mutant ∆*clr3* strain. On the other hand, epigenetic modulation was studied using suberoylanilide hydroxamic acid (SAHA), a histone deacetylase inhibitor, and nicotinamide. These treatments also resulted in reduced secondary metabolite biosynthesis. Together, these findings suggest that *clr3* plays a key role in the regulation of secondary metabolism in *P. brasilianum*.

By growing *Penicillium variabile* on a maltose medium in the presence of 5-azacytidine (a DNA methyltransferase inhibitor), varitatin A synthesis was induced [183]. In addition, by growing it on a potato-based medium in the presence of SAHA, seven polyketides were induced, including three known wortmannilactones (E, F, and H), as well as new varilactones (A-B) and wortmannilactones (M-N) [184]. In cultures treated with 50 μM of 5-azacytidine, *Penicillium citreonigrum* formed exudates, which are droplets rich in primary and secondary metabolites, inorganic substances, and proteins/enzymes and are known as guttates. These exudates were very rich in different compounds compared to the control. Indeed, 5-azacytidine induced the formation of six azaphilones (fungal metabolites with diverse biological activities), pencolide, and two new meroterpenes [185]. The addition of 5-azacytidine to the culture medium of *Penicillium funiculosum* also altered the metabolic profiles of this fungus [186]. Two new prenyleudesmane diterpenoids were extracted from the culture and exhibited cytotoxic and antibacterial activities. *Eupenicillium* sp. LG41, an endophytic fungus, was exposed to an epigenetic modulation using nicotinamide, a NAD^+^-dependent HDAC inhibitor [187]. This led to the production of many compounds: eupenicinicols C and D, along with eujavanicol A and eupenicinicol A. El-Hawary et al. [188] showed that cultures of a marine-derived strain of *Penicillium brevicompactum* exposed to nicotinamide and sodium butyrate result in the production of phenolic metabolites. In the presence of nicotinamide, many compounds, including *p*-anisic acid, benzyl anisate, syringic acid, and sinapic acid, were isolated and identified. Sodium butyrate also enhanced the production of anthranilic acid and ergosterol peroxide.

In one of the many studies to explore compounds with innovative structures and biological activities from endophytes of ancestral Chinese medicine, Guo et al. [189] used chemical epigenetic manipulation to evaluate the secondary metabolism of the *Penicillium herquei* strain, recovered from the fruiting body of *Cordyceps sinensis*. This latter has been used for thousands of years by the Chinese to boost longevity, endurance, and vitality. The DNA methyltransferase inhibitor, 5-aza-2-deoxycytidine, affected the production of secondary metabolites, purifying three previously unpublished polyketides with a pyran-2-one scaffold.

Ying et al. [190] showed that cultures of *Penicillium* sp. HS-11, isolated from the medicinal plant *Huperzia serrata*, produced two compounds in the presence of SAHA: 4-epipenicillone B and (R)-(+)-chrysogine, which are both absent under normal laboratory conditions.

The addition of 500 μM of suberoyl bis-hydroxamic acid, a Zn(II)-type or NAD^+^-dependent HDAC inhibitor, and 100 μM of nicotinamide (an NAD^+^-dependent HDAC inhibitor) to a culture of *Penicillium* sp. isolated from leaves of *Catharanthus roseus* improved the production of citreoviripyrone A and citreomontanin. In addition, nicotinamide enhanced the production of (−)-citreoviridin [191]. Xiong et al. [192] explored the role of the high-mobility group box protein, **PoxHmbB**, involved in chromatin organization and identified in *P. oxalicum*. The authors observed that conidiation and hyphae growth were delayed in a mutant PoxΔ*hmbB* strain. Pox*hmbB* regulated the expression of genes encoding plant biomass-degrading enzymes and other genes involved in conidiation. Although the suppression of the orthologous gene resulted in an absence of sterigmatocystin production in *A. nidulans* [193], the involvement of this protein in the secondary metabolism of *Penicillium* has not yet been investigated.

Tannous et al. [194] evaluated the involvement of the epigenetic reader SntB in the pathogenicity and secondary metabolism of *P. expansum*. Firstly, the results showed that the deletion of *sntB* caused numerous phenotypic changes in the plant pathogen. In the absence of *sntB*, *P. expansum* showed delayed vegetative growth, reduced conidiation, an accelerated germination rate, and decreased virulence in apples. Secondly, the data showed that *sntB* played a key role in regulating secondary metabolism, especially patulin and citrinin biosynthesis. In addition, the role of *sntB* in the positive regulation of three TFs of secondary metabolism and virulence (LaeA, CreA, and PacC) was demonstrated. Finally, this study revealed the downregulation of *sntB* in response to environmental factors such as low temperature and high CO_2_ levels, conditions to which apples are subjected during storage. These findings suggest a possible method for integrating these epigenetic control strategies to fight post-harvest fruit rot.

Finally, the chromatin regulation of small molecule gene clusters allowed the specific control of secondary metabolism gene clusters and permitted filamentous fungi to modify chemical diversity and successfully exploit environmental resources. Epigenetic regulation is considered a promising strategy for investigating unknown secondary metabolite clusters, particularly because under certain laboratory culture conditions, many clusters can remain silent, making it difficult to elucidate their functions and regulatory mechanisms [195].

## 3. Conclusions

Fungal secondary metabolism is very broad, and this review focused on metabolism regulated in the *Penicillium* genus. Given the diversity of secondary metabolites, their key roles as virulence and pathogenicity factors, and their great medical and agricultural interest, further research should be conducted on these metabolites. This review highlighted how the production of these secondary metabolites is controlled and regulated. It discussed the different levels of regulation of secondary metabolites, including specific regulators, global TFs, transduction signaling pathways, and epigenetic regulation, as well as the combination of many different parameters affecting the biosynthesis pathways of metabolites. Many TFs that affect the expression of genes involved in secondary metabolism seem to belong to the category of zinc-binding proteins. LaeA and the velvet complex proteins are considered to be global regulators and are able to control many clusters at the same time. Although much is known about these global TFs and their regulatory proteins, more research is needed to explore the details that link them to the transcription of genes involved in secondary metabolite biosynthetic pathways. This would help us to better understand the molecular mechanisms underlying this complex regulatory network. The analysis of a large number of works related to secondary metabolism regulation in filamentous fungi revealed great complexity. This complexity is suggested by the observation of inter-species differences in the impact of a given TF gene deletion on the same biosynthetic pathway.

The study of the regulation of secondary metabolite biosynthesis in *Penicillium* is much less advanced than that in *Aspergillus*, and some orthologous genes already studied in *Aspergillus* (including *rtfA*, *cpsA*, *rmtA*, *mtfA*) should be investigated in *Penicillium* sp.

## Figures and Tables

**Table 1:** Examples of identified Zn(II)_2_Cys_6_ and leucine zipper transcription factor (TF) involvement in secondary metabolism in fungi; adapted and updated from Yin and Keller [46].

TF	Biosynthetic Gene Cluster	TF Family	Species	References
AflR	Aflatoxin/Sterigmatocystin	Zn(II)_2_Cys_6_	*Aspergillus flavus* *Aspergillus nidulans* *Aspergillus parasiticus*	[51,52,53,54,55]
AsaR	Aspergillic Acid	Zn(II)_2_Cys_6_	*Aspergillus flavus*	[56]
GliZ	Gliotoxin	Zn(II)_2_Cys_6_	* Aspergillus fumigatus * * Penicillium lilacinoechinulatum *	[57,58]
XanC	Xanthocillin	Basic Leucine zipper	*Aspergillus fumigatus*	[59]
FapR	Fumagillin/Pseurotin	Zn(II)_2_Cys_6_	*Aspergillus fumigatus*	[60]
ZEB2	Zearalenone	Basic Leucine zipper	*Fusarium graminearum*	[61]
SimL	Cyclosporine	Basic Leucine Zipper	*Tolypocladium inflatum*	[62]
OtaR1	Ochratoxin A	Basic Leucine zipper	* Aspergillus carbonarius * *Aspergillus ochraceus Aspergillus westerdijkiae Penicillium nordicum*	[63]
SirZ	Sirodesmin PL	Zn(II)_2_Cys_6_	* Leptosphaeria maculans *	[58]
MlcR	Compactin	Zn(II)_2_Cys_6_	* Penicillium citrinum *	[64]
Bik5	Bikaverin	Zn(II)_2_Cys_6_	* Fusarium fujikuroi *	[65]
DEP6	Depudecin	Zn(II)_2_Cys_6_	* Alternaria brassicicola *	[66]
ZFR1 FUM21	Fumonisin	Zn(II)_2_Cys_6_	* Fusarium verticillioides *	[67,68]
CTB8	Cercosporin	Zn(II)_2_Cys_6_	* Cercospora nicotianae *	[69]
GIP2	Aurofusarin	Zn(II)_2_Cys_6_	* Gibberella zeae *	[70]
CtnA	Citrinin	Zn(II)_2_Cys_6_	*Monascus purpureus* *Monascus ruber* *Penicillium expansum*	[40,41,71]
LovE	Lovastatin	Zn(II)_2_Cys_6_	* Aspergillus terreus *	[72,73]
ApdR	Aspyridone	Zn(II)_2_Cys_6_	* Aspergillus nidulans *	[74]
CtnR	Asperfuranone	Zn(II)_2_Cys_6_	* Aspergillus nidulans *	[75]
MdpE	Monodictyphenone/ Emodin Analogs	Zn(II)_2_Cys_6_	* Aspergillus nidulans *	[76]
Cmr1p	Melanin	Zn(II)_2_Cys_6_	* Colletotrichum lagenarium *	[77]
Pig1p	Melanin	Zn(II)_2_Cys_6_	* Magnaporthe grisea *	[77]
GsfR1	Griseofulvin	Zn(II)_2_Cys_6_	* Penicillium griseofulvum *	[78]
MokH	Monacolin K	Zn(II)_2_Cys_6_	* Monascus pilosus *	[79]
CalC	Calbistrin	Zn(II)_2_Cys_6_	* Penicillium decumbens *	[42]
CnsN	Communesins	Zn(II)_2_Cys_6_	* Penicillium expansum *	[80]
Orf2	Varicidin A and B	Zn(II)_2_Cys_6_	* Penicillium variabile *	[81]
Orf10	PR-Toxin	Zn(II)_2_Cys_6_	*Penicillium chrysogenum* *Penicillium roqueforti*	[82,83]
MacR	Macrophorin	Zn(II)_2_Cys_6_	* Penicillium terrestris *	[84]
PatL	Patulin	Zn(II)_2_Cys_6_	* Penicillium expansum *	[85]
SorR1 SorR2	Sorbicillin	Zn(II)_2_Cys_6_	* Penicillium chrysogenum *	[86]
TqaK	Tryptoquialanines	Basic leucine zipper	* Penicillium aethiopicum * * Penicillium digitatum *	[87,88]
Sol4	Solanapyrone	Zn(II)_2_Cys_6_	*Ascochyta rabiei*	[89]
RolP	Leucinostatin	Zn(II)_2_Cys_6_	*Paecilomyces lilacinus*	[90]

## Abbreviations

BGC	Biosynthetic gene cluster
bZIP	Basic leucine zipper
CRP	Central regulatory pathway
DMATS	Dimethylallyl tryptophan synthase
ETP	Epipolythiodioxopiperazine
HDAC	Histone deacetylase
HOG	High osmolarity glycerol
NLS	Nuclear localization sequence
NRPS	Non-ribosomal peptide synthetase
OTA	Ochratoxin A
PKS	Polyketide synthase
SAHA	Suberoylanilide hydroxamic acid
TAP	Tandem Affinity Purification
TC	Terpene cyclase
TF	Transcription factor
WT	Wild Type
